# P-1240. Optimal Vancomycin Model Selection for Obese Patients Receiving Outpatient Parenteral Antimicrobial Therapy (OPAT)

**DOI:** 10.1093/ofid/ofae631.1422

**Published:** 2025-01-29

**Authors:** Kimberly A Couch, Quyen Luu, Richard C Prokesch, John S Adams, Joseph F John, Lucinda J Van Anglen

**Affiliations:** Healix Infusion Therapy, LLC, Sugar Land, Texas; Central Georgia Infectious Disease Associates, LLC, Macon, Georgia; Infectious Diseases Associates, PC, Riverday, Georgia; Knoxville Infectious Disease Consultants, Knoxville, Tennessee; Low Country Infectious Diseases, PA, Charleston, SC; Healix Infusion Therapy, LLC, Sugar Land, Texas

## Abstract

**Background:**

Optimal OPAT dosing of vancomycin (VAN) is challenging in obese patients. Multiple Bayesian pharmacokinetics (PK) models to aid in AUC estimation exist, including several obesity-specific models. PK population models in the absence of drug serum levels (*a priori*) are useful to initiate therapy, but once drug serum levels are obtained, model performance is based upon individual patient PK (*a posteriori*). This study is the first evaluation of the optimal PK model for obese patients receiving OPAT.

**Figure:**
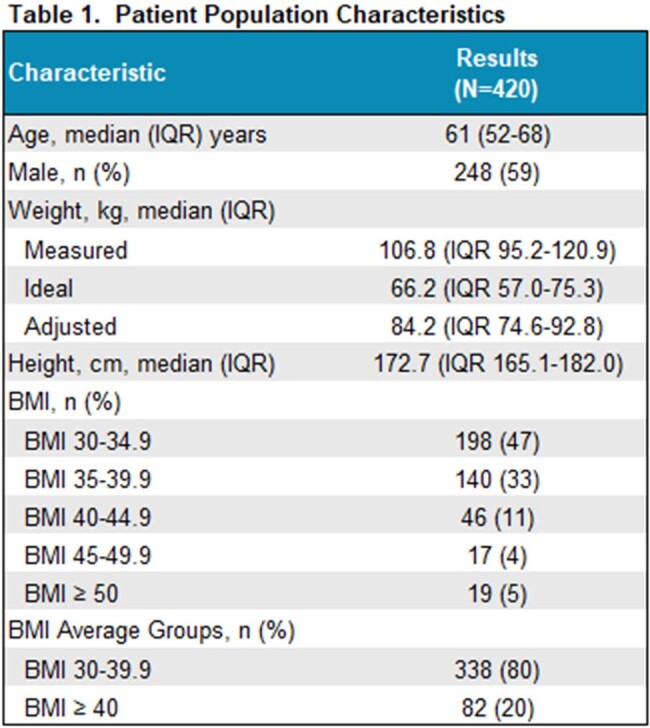

**Methods:**

Analysis included all OPAT patients who received VAN in 2022-2023 with at least one therapeutic drug level, and a body mass index (BMI) ≥ 30 kg/m^2^. Data collection included demographics, anthropometrics, treatment information, laboratory values, and AUC_24_. Ten PK models were used to predict VAN serum concentrations and calculate AUC24 using a Bayesian dosing software. The performance of each model was assessed using *a priori* root mean square error (RMSE) and *a posteriori* RMSE for each model. The average RMSE was also calculated for patients with BMI 30-39.9 kg/m^2^ and for those ≥40 kg/m^2^. The model with the best performance for each BMI category was reported.

**Figure:**
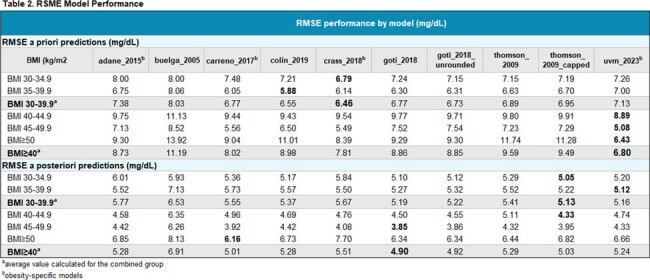

**Results:**

Of 920 VAN OPAT patients, 420 (45.6%) were identified with a BMI ≥30 kg/m^2^. Population characteristics are included in Table 1, with 80% having a BMI 30-39.9 kg/m^2^. Median duration of VAN was 23 days (IQR: 12-35). The RMSE calculations per model and BMI groups are shown in Table 2. Best model fit varied among BMI groups and is indicated in bold type. For RMSE *a priori*, the Crass model performed the best for BMI 30-39.9 kg/m^2^ and the UVM model was best for BMI ≥40 kg/m^2^. The RMSE *a posteriori* with best performance for BMI 30-39.9 kg/m^2^ was Thompson capped, with UVM and Goti also performing well. For BMI ≥40 kg/m^2^, RMSE *a posteriori*, the Goti model was the best performing.

**Conclusion:**

Obesity models provided good performance for *a priori predictions* and performed similarly to non-obese models in *a posteriori* predictions. This research supports use of both obesity and non-obesity models for VAN PK in OPAT.

**Disclosures:**

**Joseph F. John, Jr., MD, FACP, FIDSA**, MicroGenDx: Advisor/Consultant **Lucinda J. Van Anglen, PharmD**, Cumberland Pharmaceuticals: Grant/Research Support|Ferring Pharmaceuticals: Grant/Research Support|Novartis Pharmaceuticals: Grant/Research Support|Takeda Pharmaceuticals: Grant/Research Support

